# Natural Diatom Biosilica as Microshuttles in Drug Delivery Systems

**DOI:** 10.3390/pharmaceutics11100537

**Published:** 2019-10-15

**Authors:** Joachim Delasoie, Fabio Zobi

**Affiliations:** Department of Chemistry, Fribourg University, Chemin du Musée 9, 1700 Fribourg, Switzerland; joachim.delasoie@unifr.ch

**Keywords:** diatom, drug delivery, biosilica, composite, natural, surface functionalization

## Abstract

Unicellular diatom microalgae are a promising natural resource of porous biosilica. These microorganisms produce around their membrane a highly porous and extremely structured silica shell called frustule. Once harvested from living algae or from fossil sediments of diatomaceous earth, this biocompatible and non-toxic material offers an exceptional potential in the field of micro/nano-devices, drug delivery, theranostics, and other medical applications. The present review focused on the use of diatoms in the field of drug delivery systems, with the aim of presenting the different strategies implemented to improve the biophysical properties of this biosilica in terms of drug loading and release efficiency, targeted delivery, or site-specific binding capacity by surface functionalization. The development of composite materials involving diatoms for drug delivery applications is also described.

## 1. Introduction

**Figure 1:**
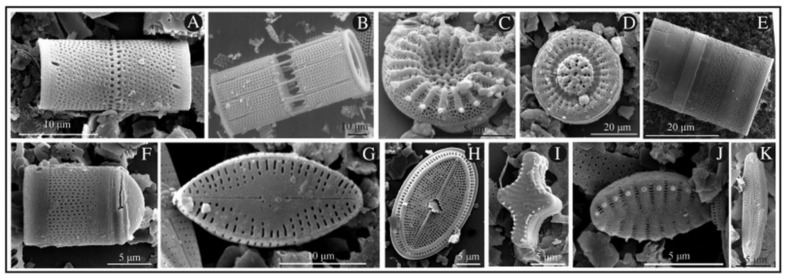
Common diatom species. (**A**) *Aulacoseira ambigua*, (**B**) *Aulacoseira granulata*, (**C**) *Stephanodiscus cf. minutulus*, (**D)** *Discostella stelligera*, (**E**) *Melosira undulata*, (**F**) *Orthoseira dendroteres*, (**G**) *Diadesmis confervacea*, (**H**) *Cocconeis placentula*, (**I**) *Staurosira construens*, (**J**) *Staurosirella pinnata*, (**K**)* Achnanthidium minutissimum*. Reprinted from [16], copyright (2017), with permission from Elsevier.

Starting in 1950, the use of drug carriers to vehicle pharmaceutical active ingredients (API) in the body gained a rapid interest in the field of therapeutics [1]. Before this period, drugs were generally compressed into pills and administrated as therapeutics that did not allow for controlled, targeted, or sustained release. The development of material able to shuttle API in the human body with controlled kinetics and targeting capacity has become an objective of great importance since then. Among the materials eligible as microshuttles (i.e., materials composed of micrometer size particles capable of drug delivery), both natural or synthetic mesoporous silica-based materials have gained rapid interest in the field over the last decades [2,3,4,5,6,7,8]. Indeed silica micro/nano-particles present several benefits compared to other materials due to the ease of particle size control during their synthesis, their chemical inertness, high surface porosity, flexible surface modification, thermal stability, and biocompatibility [2,9,10,11,12,13]. However, the fabrication of synthetic mesoporous silica requires advanced skills, often involves the use of toxic chemicals, and results in the formation of non-reusable polluting byproducts, leading to a poor cost-effective process. To overcome this problem, 20 years ago Morse proposed an alternative to the synthetic production of mesoporous silica by using naturally occurring diatoms [14]. Indeed, diatom microalgae, which are unicellular microorganisms living in either fresh or seawater all over the world, build solid silica shells around their membrane to protect them from environmental stress. There are at least 30,000 and probably three times more different diatom species in existence, each characterized by different forms and structures of their outer silica shell (Figure 1) [15,16].

This silica shell, made of silicon dioxide and called “frustule”, represents a fantastic and inexpensive source of highly structured mesoporous silica [17,18,19]. As outlined in the following sections, diatoms are ideal micro/nano-carrier materials, as they permit retention of the nature and bioactivity of the loaded drug, while being capable of carrying it to the site of action. Thus, the chemical characteristics, various available morphologies, specific size and porosity, tunable surface functionalization, biocompatibility, and inertness of diatoms make frustules harvested from microalgae a unique tool in the field of drug delivery applications [20,21,22,23,24]. Furthermore, the particular morphologies of diatoms and properties such as large surface area, wide porosity, and highly organized hierarchical structure present valuable features in the field of drug delivery. Their biomineralization process has also been taken as an inspirational model for the elaboration of synthetic materials and efficient drug carriers [25]. Véliz and coworkers synthetized an insulin delivery system encaged in a larger diatom growth-guided calcium carbonate skeleton [26]. Synthetic mesoporous silica similarly exhibits surface mineralization potential, acting as template tailoring the formation of complex structures [27,28]. The diatom frustule complex hierarchical structure has inspired various research studies in a broad range of different fields such as medical implantable devices, nanoimprint lithography (NIL), or bio-inspired deformation element [29,30,31]. For example, in the field of photonic devices, Li et al. reported the fabrication of metamaterial absorbers directly inspired by the hierarchical structure of diatom frustules. Their study demonstrated the enhanced properties of a new structure exhibiting broad band high-absorption properties [32]. For all these applications, as well as the ones we describe in the following pages, this natural biomaterial can be recovered in two ways: through cultivation, harvest, and isolation of frustules from living diatoms, or through mining diatomaceous earth, a fossil sediment enriched in silicon diatom frustules [33]. Although cultivating algae in a laboratory could be a challenging process, diatomaceous earth is the most abundant resource of silicon dioxide in the world; hence, it is a very inexpensive and eco-friendly starting material for the fabrication of theranostic devices and drug delivery systems [34]. Diatomite was recognized as safe by the FDA for human consumption when used as adjuvant or carrier in different forms of preparation (Code of Federal Regulations (CFR), Title 21, Sections 177.2410, 178.3297, 182.90, or 184.1420), but it still requires approval for use in the pharmaceutical industry and in medicine.

## 2. Diatoms as a Natural Biocompatible Material for Therapeutic Applications

**Figure 2:**
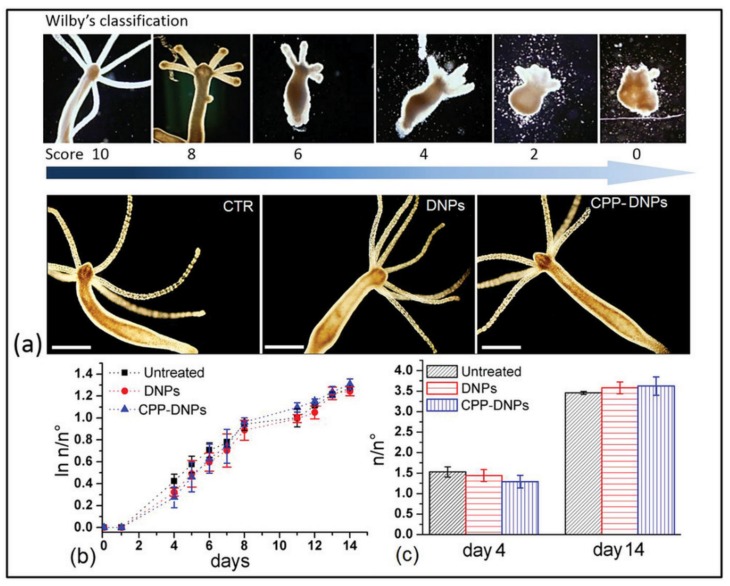
In vivo effects of diatomite nanoparticles (DNPs) on *Hydra* morphology and growth rate: (**a**) upper panel: Wilby’s classification of *Hydra* morphological alterations due to exposition to a toxic environment; lower panel: representative images of living *Hydra* polyps, untreated CONTROL (CTR) and treated with bare DNPs and cell penetrating peptide (CPP)-modified DNPs (CPP-DNPs) up to 72 h; scale bars, 500 µm. (**b**) Graph showing ln n/n^0^ values at each time point, where n is the total number of polyps and n0 is the number of founder forms. (**c**) Graph showing the n/n^0^ ratio (s.d.) obtained from growth curves at day 4 and 14. Error bars represent s.d. (*n* = 3). Reproduced from [45], copyright (2019), with permission from John Wiley and Sons.

Several different in vitro studies have demonstrated the biocompatibility and non-toxicity of silica nanoparticles (NPs) [35,36,37,38,39,40]. Diatom frustules made of pure silica also satisfy this statement, making them a suitable alternative to the fabrication of micro-devices for medical applications. In 2016, after fully characterizing the covalent modification of *Thalassiosira weissflogii* frustules with (3-Aminopropyl)triethoxysilane (APTES) or (3-Mercaptopropyl)trimethoxysilane (MPTMS), Cicco and co-workers demonstrated the prompt adhesion and proliferation of normal human dermal fibroblasts (NHDF) and sarcoma osteogenic (Saos-2) cells on surfaces coated with bare diatoms [41]. The authors showed that surfaces coated with MPTMS-diatoms improved vitality and shape of the two cells lines compared to surfaces coated with simple bare diatoms. On the other hand, on surfaces coated with APTES-diatoms, the external amino groups negatively affected both the adherence and proliferation of the two cell lines, consistent with previously reported results on interactions of cell surfaces with cationic nanoparticles [42,43,44]. While surface coating with diatoms plays a crucial role in tissue integration, it was soon recognized that natural biosilica from diatoms also offers a promising biocompatible material for medicinal applications. In early 2019, Terracciano et al. reported the in vivo compatibility and non-toxicity of diatom nanoparticles using a small invertebrate, the cnidarian freshwater polyp *Hydra vulgaris*, as an animal model [45]. *Hydra* polyps are highly sensitive to both organic and inorganic pollutants in their environment, leading in most cases to delayed growth, morphological changes, induction of apoptosis, and even alteration of gene expression [46,47]. The Wilby’s classification (Figure 2a) allowed for the estimation of the morphological change in polyps on a scale of 10 (where 10 relates to a healthy specimen and 0 to a completely disintegrated individual). No significant difference in morphology or growth rate was revealed after incubation of *Hydra vulgaris* with concentration up to 3.5 g/L over 3 days of either bare diatomite nanoparticles (DNPs) or cell penetrating peptide (CPP)-modified DNPs (CPP-DNPs) (Figure 2b,c) when compared to untreated populations. Confocal fluorescence experiments on DAPI dye stained nuclei showed no significant cell apoptosis induced by internalized CPP-DNPs, further strengthening the hypothesis of DNPs innocuity towards living organism. In addition, the increased internalization of CPP-DNPs compared to bare DNPs was also demonstrated in this study by in vivo fluorescence microscopy analysis.

In terms of possible administration routes of diatom-loaded drugs, oral administration is the most obvious form. Oral administration is a common and convenient way to deliver therapeutics in the body, and it is generally limited to small molecules as pharmaceuticals that are more complex could be easily degraded through digestion in the stomach. Parenteral administration regroups the routes involving injections, as intravenous (IV) or intramuscular (IM) administrations, offering the advantage of targeted effect and reduced toxicity when compared to other routes of delivery [48,49]. The ideal size of particles in a suspension to be administrated by injection should range between 100 to 300 nm, a scale enhancing their permeability and retention effect in solid tumors [50]. Diatoms are used as a food additive and could be safely administrated through the oral route; however, their size should be decreased in a controllable manner to be eligible for parenteral administration. Fine grinding and ball milling techniques allow for the reaching of the specific nanometric scale required for injectable nanosuspension. A possible drawback to the use of raw diatoms in therapeutic formulations is the poor degradability of silica in biological fluids [11,51,52]. Synthetic porous silicon (pSi), sponge-like structures made of monocrystalline silicon, have been demonstrated as being fully biodegradable. Recently, Bao et al. converted diatom frustules to pure silicon diatom frustule replicas by magnesio-thermic reduction [53,54]. This conversion of diatom silica to pure silicon presents the advantages of retaining their highly porous and structured morphology while becoming of good biodegradability. Maher et al. conducted studies on these diatom frustule silicon replicas for drug delivery and medical applications (vide infra) [55,56].

## 3. Diatoms as Drug Carriers

**Figure 3:**
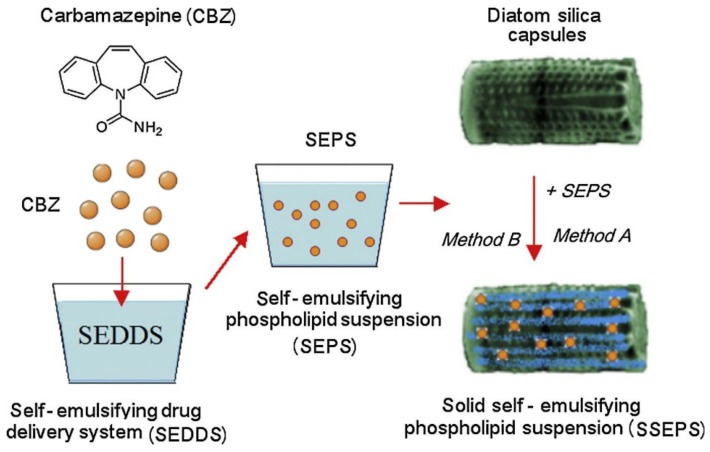
Schematic presentation of solid self-emulsifying phospholipid suspension (SSEPS) preparation. CBZ—carbamazepine; SEDDS—solid self-emulsifying drug delivery system; SEPS—self-emulsifying phospholipid suspension; diatoms—diatom silica particles. Reprinted from [58], copyright (2014), with permission from Elsevier.

In the last decade, diatoms have been increasingly recognized as a promising biomaterial tool for drug delivery applications. In early 2011, Aw and co-workers demonstrated the feasibility of loading diatoms with a poorly water-soluble drug model, the nonsteroidal anti-inflammatory drug (NSAID) Indomethacin, with a loading efficiency up to 22 wt% (percentage by weight) [57]. They measured an important initial fast drug release during the first 6 h assigned to the drug molecules physisorbed at the outer surface of the diatoms that are driven by diffusion mechanisms. A secondary and more sustained drug release was then observed over a 2 week period and was attributed to the drug siting into the pores and the hollow structures of the diatoms. In 2013, Zhang et al. studied the sustained release and permeation enhancement of mesalamine and prednisone by diatom microparticles in the gastro-intestinal tract [40]. They demonstrated very low cytotoxicity of bare diatoms at a concentration of up to 1000 µg/mL on colonic cancer cell lines Caco-2, HT-29 and HT-116. In vitro studies showed prolonged sustained release of the drugs in different body-like fluids, mimicking the gastro-intestinal pathway. Furthermore, in Caco-2/HT29 monolayers, diatom microparticles enhanced drug permeation by shuttling both drug models used in the study. The mechanism by which diatoms promote paracellular permeation enhancement occurs via opening the intercellular tight junctions, as confirmed by transepithelial/transendothelial electrical resistance (TEER) experiments. In 2014, Milović and co-workers focused on the combined use of solid self-emulsifying phospholipids suspension (SEPS) with diatoms as a new drug formulation [58]. For this purpose, carbamazepine (CBZ), an anticonvulsant operating as a sodium channel blocker in cell membranes, was chosen as a poorly soluble drug model. The higher dissolution rate of diatoms loaded with CBZ SEPS mixtures, either physically mixed or simply adsorbed on diatoms, was demonstrated (Figure 3). Moreover, the authors showed that a physical mixture of CBZ SEPS and diatoms provided long-term stability and maintenance of the pharmaceutically acceptable P-monoclinic form of CBZ without loss of the enhanced dissolution rate properties. The study was the first example of the combination of self-emulsifying drug delivery system (SEDDS) with diatoms as solid biocompatible nano-carriers, having the aim of developing innovative pharmaceutical formulations.

**Figure 4:**
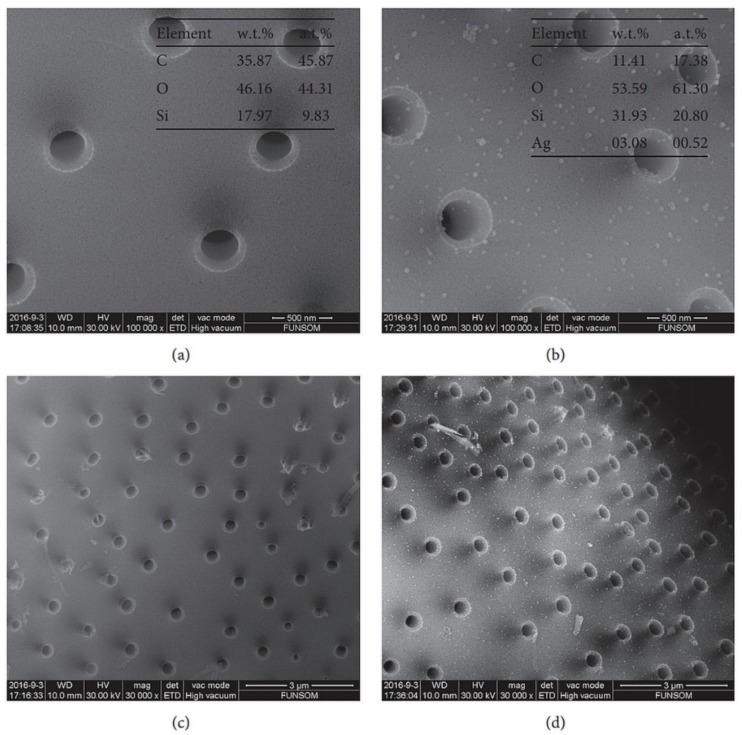
Scanning electron microscopy (SEM) images at different magnifications: (**a**,**c**) of the diatomite; (**b**,**d**) of the nanosilver-modified diatomite (nAgDT). Corresponding energy-dispersive X-ray spectroscopy (EDS) results are shown in (**a**,**b**). Reproduced from [61], copyright (2018), Hindawi, licensed under CC BY 4.0.

In 2014, Gnanamoorthy et al. investigated the in vitro drug loading and release of the well-known antibiotic streptomycin from the cylindrical *Coscinodiscus concinnus* diatom frustules [59]. The enhanced drug loading and release properties of acid-treated diatoms compared to bare diatoms were highlighted. The acid treatment led to well hydroxylated surfaces, explaining the enhancement of diatom drug delivery properties. With loading capacities of more than 30 wt%, Gnanamoorthy et al. reported a typical two phase release of the drugs—an initial burst release over 6 h and a more sustained release up to 7 days. Similarly, Vona et al. used diatoms as biocarriers for Ophiobolin A, a naturally occurring anticancer molecule produced by fungi [60]. They outlined the increased drug loading and release properties of diatoms after acid and peroxide treatments, which increased silanol groups at the diatoms surface, thus increasing the percentage of drug encapsulation (or drug loading (DL) wt%) from 0.47 to 9.47 wt%. More recently, Sun et al. produced nanosilver-modified diatomite (nAgDT) by directly mixing in situ silver ions with ammonia solution, pure diatomite, Tollen’s reagent, and glucose [61]. The well-loaded nanosilver particles (3.08 wt% of silver in the nAgDT) were equally distributed on the diatomite (Figure 4).

Cytocompatibility tests on NIH3T3 fibroblasts and MC3T3-E1 osteoblasts showed that the formulation is not toxic at a concentration under 1 mg/mL, whereas antibacterial activities evaluated with both *Escherichia coli* and *Staphylococcus aureus* bacteria showed efficiency at doses over 0.5 mg/mL in the case of *E.coli* and 0.1 mg/mL in the case of *S.aureus*. These results outlined the potential range of application for this new material in the field of bone and orthopedic implants.

## 4. Functionalized Diatoms: Smart Targeting Vehicles for Drug Delivery

**Figure 5:**
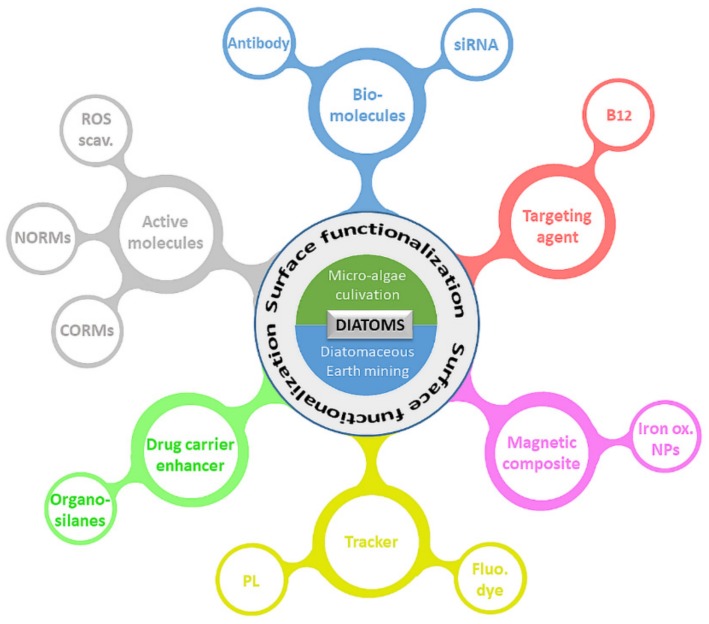
Overview of the potential surface modification of diatoms used in pharmaceutical applications. NP: nanoparticles. Small interfering RNA (siRNA); Vitamin B_12_ (B_12_); Iron oxide nanoparticles (Iron ox. NPs); Fluorescent dye (Fluo. dye); Photoluminescent compound (PL); Carbon monoxide-releasing molecules (CORMs); Nitric oxide releasing molecules (NORMs); Scavengers of reactive oxygen species (ROS scav.).

In the last two decades, efforts have been made to investigate and develop innovative drug delivery systems on the basis of diatoms [2,9,10,11,12,13,62]. Several strategies leading to enhanced diatom drug carriers have been presented, and Figure 5 summarizes the numerous possibilities currently available for diatom surface modifications and functionalization.

The ability to be functionalized with different components, together with a high drug-loading capacity, make diatoms a powerful and promising tool in pharmaceutics. Drug loadings are generally achieved by soaking the diatoms into a concentrated solution of selected drugs. As a result, in most cases, the drugs are physisorbed or chemisorbed onto surfaces and further released by concentration gradient-driven diffusion. In other cases, such as composites and compacted pills, the drug release is mainly driven by dissolution or degradation of the pharmaceutical formulation. Table 1 summarizes some surface functionalization and drug loading efficiencies from different studies carried out in the field.

### 4.1. Diatoms Functionalized via Organosilane Coating

**Figure 6:**
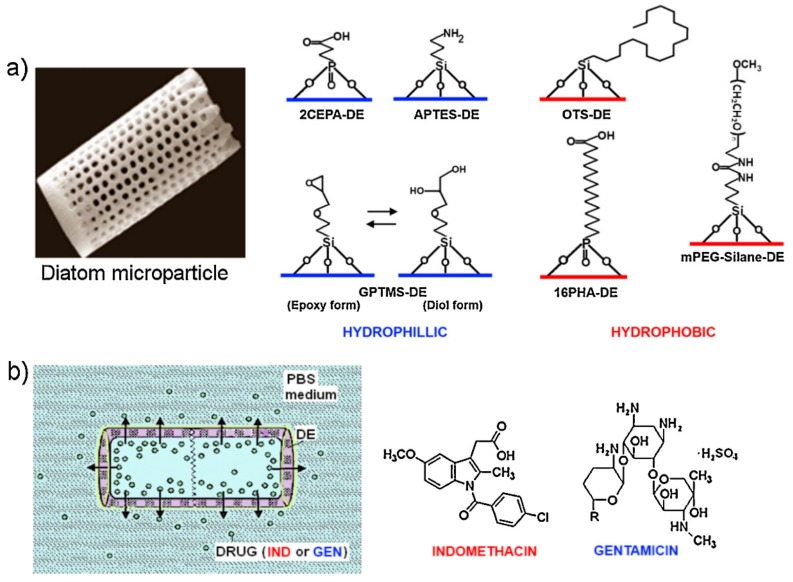
Schematic diagram showing: (**a**) structure of diatomaceous earth (DE) microparticles along with surface functionalization using organosilanes (APTES, GPTMS, OTS and mPEG-Silane) and phosphonic acid (2-CEPA and 16-PHA) to render DE surface either hydrophilic or hydrophobic; (**b**) scheme of the drug release from DE structure with chemical structure of the two model drugs IND—hydrophobic and GEN—hydrophilic used in this study. Reprinted from [64], copyright (2012), with permission from Elsevier.

In 2008, Townley et al. reported the tethering of *Coscinodiscus wailesii*, a large centric diatom, with different antibodies, using either the amino groups or the sugar moieties of the antibodies to bind the diatom surface [74]. The authors demonstrated the potential application of the system in immunoprecipitation assays by tethering diatoms to tubulin antibodies and selectively isolating them from total *Drosophila* homogenate. In 2012, Bariana et al. presented diatom microparticles functionalized with different hydrophilic or hydrophobic organosilanes and phosphoric acids (Figure 6 and Table 1). Their studies focused on the loading and release capacities of the modified diatoms towards two common therapeutics, the NSAID Indomethacin and the antibiotic Gentamicin [64]. They demonstrated that the hydrophilic coating of the diatoms leads to an increased loading capacity and slower release of the hydrophobic Indomethacin, whereas the hydrophobic coating of diatoms promotes the same behavior with the hydrophilic drug Gentamicin. In the latter case, however, a lower drug loading and faster release was observed. 

**Figure 7:**
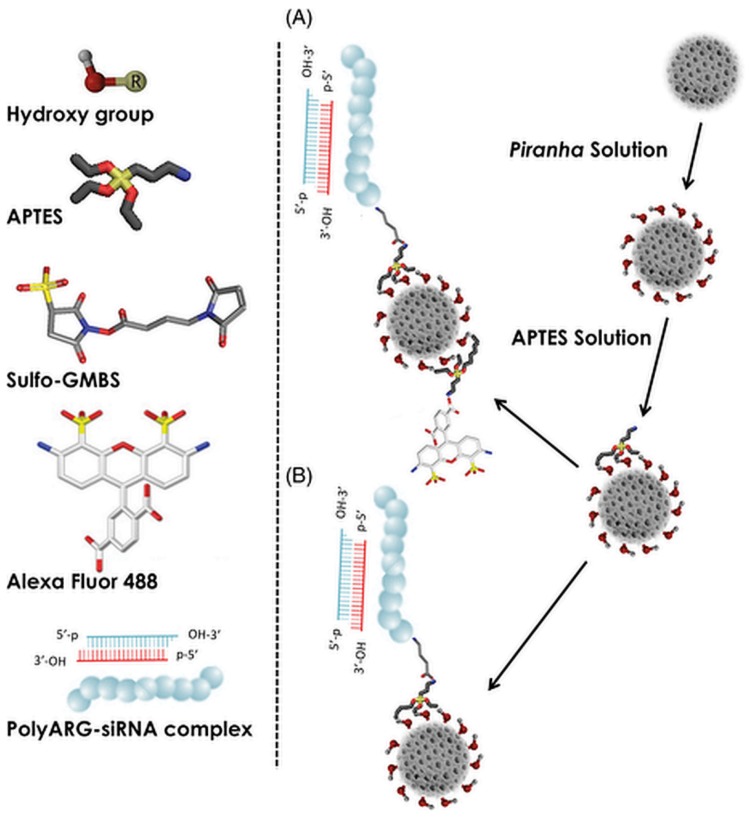
Chemical surface modification of DNPs. (**A**) Labeled siRNA-loaded DNPs and (**B**) label-free siRNA-loaded DNPs. Reproduced from [75], copyright (2017), with permission from John Wiley and Sons.

This study further confirmed the great potential and the tunability of drug delivery systems made with the inexpensive and eco-friendly nanoporous biosilicas from diatoms. In 2013, Kumeria et al. reported the functionalization of diatoms with graphene oxide (GO) [65], a modification that led to the enhancement of the diatoms’ photoluminescent (PL) properties. This interesting biomaterial, further exploitable in biosensing applications, shows improved Indomethacin loading capacities and a pH-dependent releasing behavior of the model drug. In 2014, Ruggiero and co-workers highlighted the well internalization of functionalized and size-reduced diatom nanoparticles (100 to 300 nm) into epidermoid cancer cells (H1355) [66]. For this purpose, the authors treated the diatoms by crushing and sonication steps to reduce their size to the nanoscale before proceeding with their functionalization with organosilane and further tetramethylrhodamine isothiocyanate (TRITC) as a traceable dye. Confocal microscopy imaging of these nanoparticles demonstrated the well internalization into cytoplasm of epidermoid carcinoma cells, reinforcing the potential of this biomaterial in anticancer delivery applications. The same year, Aw et al. presented another study on the functionalization of diatoms with different hydrophilic or hydrophobic compounds, focusing on the assessment of the drug loading and release capacities of the modified diatoms towards the water-insoluble drug Indomethacin [67]. They demonstrated that surface functionalization with *N*-[3-(trimethoxysilyl)propyl]ethylenediamine increases the loading capacity of diatoms up to 60 wt% and highlighted the correlation between active polar functional groups at the diatom surface, showing both the increased loading capacity and prolonged release property of the drug (Table 1). In 2014, Rea et al. used diatomite (earth made of the siliceous remains of diatoms) as a drug vector for the transport of siRNA inside human epidermoid cancer cells H1355 (Figure 7) [36]. To increase the cell uptake and internalization process of these diatoms, their mean size was decreased down to the nanometric scale (mean size of 200 nm). The biocompatibility and relative innocuity of the nonmetric-sized diatomite towards the H1355 cell line at a concentration up to 300 µg/mL exemplifies the potentiality of these particles in nanomedicines and drug delivery applications. Via the functionalization of diatomite with Poly D-arginine peptide/siRNA complex, Rea and co-workers showed, respectively, by fluorescence spectrometry and confocal microscopy, the in vitro sustained released and the efficient internalization of siRNA into the cytoplasm of H1355 cancer cells. Furthermore, gene silencing of specific targeted mRNA was demonstrated by western blot analysis, confirming the efficiency of their newly designed siRNA delivering system. 

**Figure 8:**
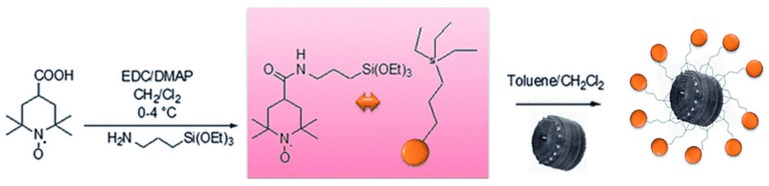
Synthesis of the TEMPO-APTES derivative and subsequent immobilization on frustule biosilica. Adapted from [68], copyright (2015), with permission from John Wiley and Sons.

In 2015, Cicco et al. focused on the use of antibiotic-loaded diatom frustules modified with a reactive oxygen species (ROS) scavenger in regenerative medicine applications (Figure 8) [68]. The design was based on the idea of delivering the efficient antibiotic ciprofloxacin (CPFX), and preventing its adverse side effects, such as oxidative damage to cells, by functionalizing the diatoms with (2,2,6,6-tetramethylpiperidin-1-yl)oxy (TEMPO) as an antioxidant and antiradical agent.

**Figure 9:**
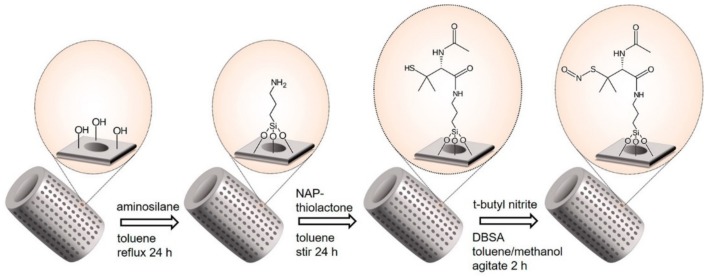
NO-releasing diatomaceous earth derivatization schematic featuring APTES as a representative silane. Reprinted with permission from [76], copyright (2017), American Chemical Society.

The authors demonstrated the efficient functionalization of their diatom frustules with TEMPO (FT) by silanization, as well as the positive effect of FT-functionalized frustules on MG63 human osteosarcoma cell viability. Moreover, the enhancement of bone cells adhesion and proliferation on glass surface covered with FT was outlined. The same year, Vasani and co-workers designed thermo-responsive biosilica microcapsules and assessed the potential of this system by testing in vitro the release of Levoflaxin, an antibiotic used to treat various bacterial infections in humans [69]. For this purpose, diatoms were functionalized with a derivatized silane-based atom transfer radical polymerization (ATRP) initiator. Alongside this, an oligo(ethylene glycol) methacrylate copolymer was prepared. Synthesis of the copolymers allowed producing compounds with unique reactivity to specific temperature changes in their environment. Activator regeneration by electron transfer based atom transfer radical polymerization (ARGET-ATRP) was the method selected to synthetize and graft the copolymers on the diatom surface. Drug release assessment above and below the lower critical solution temperature (LCST) of the grafted copolymers demonstrated a temperature-dependent release profile of the Levoflaxin in PBS at pH 7.4. To prove the retained activity of the antibiotic after its release, zone of inhibition (ZOI) studies were performed, and the results successfully confirmed this hypothesis. Terraciano et al. modified diatoms with a pegylated polymer ending with a cell-penetrating peptide (CPP) [37]. They demonstrated enhanced drug loading capacity of the poorly water-soluble anticancer sorafenib (up to 22 wt% in diatoms) and improved releasing profiles in aqueous solution and haemocompatibility (assessed by red blood cell morphological study) after 48 h incubation at 200 µg/mL with erythrocytes. The authors also showed a considerable increase of cellular uptake of the CPP-modified diatoms in two different cancer cell lines, MCF-7 and MDA-MB-231 breast cancer cells. Martucci et al. conjugated diatoms with an idiotype-specific peptide (Id-peptide) able to target the antiapoptotic factor B-cell lymphoma/leukemia 2 (Bcl2) [38]. They demonstrated a threefold increased uptake of these nanoparticles by Id-peptide recognition in A20 lymphoma cells compared to nonspecific 5T33MM myeloma cells. They also showed a more effective Bcl2 gene silencing using their modified nanoparticles as targeting vehicles when compared with the conventional lipofectamine transfection agent, suggesting the successful delivery of the siRNA into the host A20 lymphoma cells. In 2017, Managò et al. reported the internalization kinetics and the cytoplasmic localization analysis of siRNA-modified diatomite (Figure 7) in human lung epidermoid carcinoma cell line (H1355) by Raman spectroscopy [75]. Raman imaging of labelled-free siRNA-DNPs revealed efficient internalization of the nanoparticles within the first 40 h of incubation, as well as their co-localization in lipidic vesicles, leading to the conclusion that the internalization process takes place via endocytosis. The internalization kinetics and cytoplasmic localization were further confirmed by confocal fluorescence microscopy on cells treated with fluolabelled siRNA-DNPs and by PL measurements of supernatant from incubation wells. In 2017, Grommersch et al. reported the synthesis of diatomaceous earth (DE) modified with S-nitroso-N-acetyl-penicillamine (SNAP), a nitric oxide (NO)-releasing molecule (Figure 9) [76]. NO is a gasotransmitter that plays a crucial role in many different physiological processes. Its potential therapeutic effects in treating different diseases and pathophysiological processes, such as thrombosis, inflammation, vasodilation, inhibition of platelet adhesion and aggregation, neurotransmission, and immune system regulation, has been well recognized from many years [77,78,79,80].

**Figure 10:**
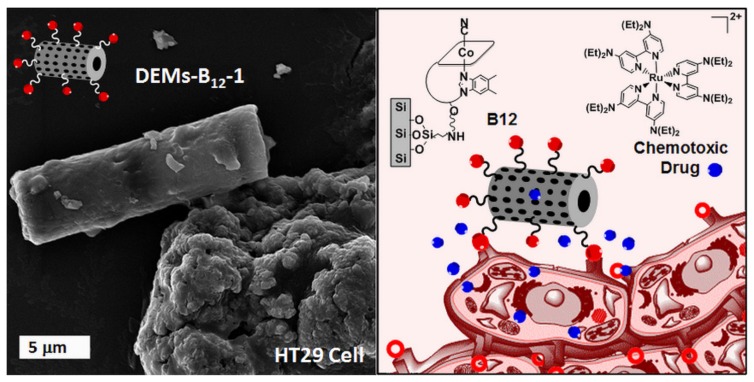
Graphical abstract representing an SEM image of a vitamin B_12_ modified diatom (DEMs-B_12_-1) anchored to a HT29 colonic cancer cell and the corresponding scheme. Reproduced from [73], by permission of The Royal Society of Chemistry.

After optimizing the synthetic pathway by testing different organosilane linkers, Grommersch and co-workers confirmed, by chemiluminescence quantification, the ability of their SNAP-DE material to sustain NO release over a period of 24 h. SNAP-DE showed antibacterial activity when tested on *S. aureus*, a common pathogen responsible for hospital-acquired infection, with bacterial reduction up to 92.95% ± 2.6%. Moreover, in vitro cytotoxicity assays on 3T3 mouse fibroblast cells revealed no toxicity of the SNAP-DE toward mammalian cells, making it a promising material in biomedical applications. Sasirekha et al. used the diatom *Amphora subtropica*-modified with chitosan as a drug vehicle for doxorubicin, a chemotherapeutic belonging to the class of anthracyclines [72]. Doxorubicin is known to provoke many side effects, and this dose-limiting toxicity encourages research of more efficient drug delivering systems. Through in vitro studies on immortalized lung cancer A549 cells, Sasirekha and co-workers demonstrated the enhanced cytotoxicity effect of doxorubicin due to sustained release from drug-loaded chitosan modified *A. subtropica* diatoms (Chi@AF) when compared to the free drug. They also proved the haemocompatibility of their doxorubicin-loaded material Chi@AF-DOX at a particle concentration up to 50 µg/mL. Recently, Delasoie et al. presented the synthesis of vitamin B_12_ modified diatoms (DEMs-B_12_-1) as a cancer cell-targeting delivery system for inorganic drugs (Figure 10) [73]. They highlighted the increased adherence of the B_12_ modified diatoms to MCF-7 breast cancer and HT29 colonic cancer cell lines when compared to unmodified diatoms. The authors also showed the resistance of the B_12_ coating to the intestinal tract fluids and the slow release of an experimental ruthenium anticancer complex in lipophilic environments at the cell membrane.

### 4.2. Diatoms Functionalized with Magnetic Coating and Antibodies

**Figure 11:**
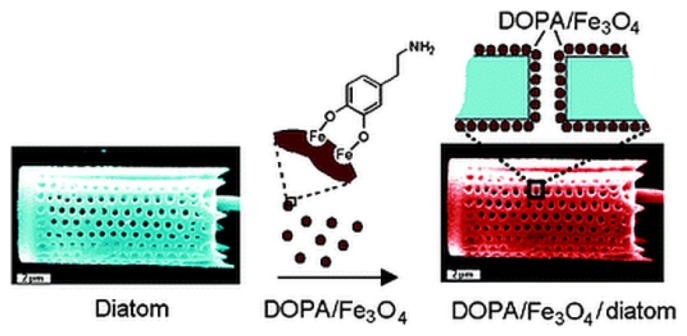
Scheme of functionalization of diatoms with dopamine-modified iron oxide nanoparticles to introduce their magnetic properties. Reproduced from [63], copyright (2010), with permission from The Royal Society of Chemistry.

In early 2010, Losic and co-workers reported the functionalization of diatoms with a dopamine iron oxide (DOPA/Fe_3_O_4_) composite [63]. A simple electrostatically driven self-assembly approach was adopted and proved efficient in functionalizing diatoms with DOPA/Fe_3_O_4_ (Figure 11). The ability of these microcarriers to be magnetically driven in solution was demonstrated by simple tests in the presence of an external magnetic field. Moreover, the free ending amino group of DOPA resulted in availability for further functionalization with biomolecules of interest. For this purpose, fluorescein isothiocyanate (FITC) was used as a model fluorophore by coupling it to the free ending amino groups immobilized on the diatoms. Finally, the loading and release properties of DOPA/Fe_3_O_4_-diatoms was assessed via the common nonsteroidal anti-inflammatory drug (NSAID) Indomethacin. This composite material is capable of partially sustaining the drug release over 2 weeks.

**Figure 12:**
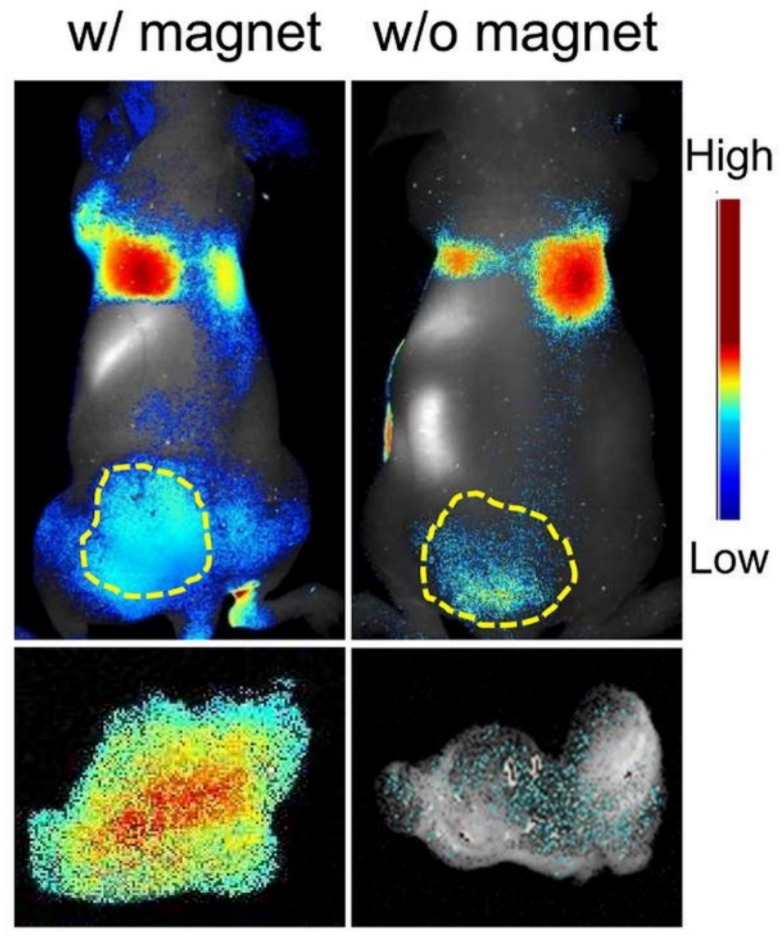
(Upper row) In vivo imaging results. Correlating with the MRI data, significantly more fluorescence signals were observed in tumors that were attached with a magnet. (Bottom row) Ex vivo imaging with dissected tumors. Compared to the controls, the researchers observed a 6.4 times higher accumulation of diatoms in the tumors that had been attached with a magnet during the process. Reproduced from [81], copyright (2014), with permission from The Royal Society of Chemistry.

Also in 2013, Todd and co-workers reported the fabrication of magnetically guidable diatom materials [81]. Human serum albumin (HSA)-coated iron oxide nanoparticles (IONPs) were loaded onto diatoms (DTMs) to confer them their magnetic properties. The 8 wt% Fe loading, determined by inductively coupled plasma (ICP) analysis, explained the important magnetic susceptibility of the IONP-DTMs. Cytotoxicity study on 4T1 murine breast cancer cell line showed good biocompatibility up to 625 µg·mL^−1^ with 80% of the cell population kept viable after 24 h exposure. Furthermore, in vivo testing was conducted on ZW800-loaded IONP-DTMs injected in animals equipped with or without a magnetic bar fixed to the skin on the site of their tumor (ZW800 is a fluorescent dye used as a drug model in the study). One hour after the intravenous injection of ZW800-loaded IONP-DTMs into 4T1 tumor xenografts, the animals were imaged by T2-weighted MR and by fluorescence. Both tests revealed a specific tumor accumulation in the magnet-treated group region of interest (ROI) analysis. After ex vivo fluorescence imaging on dissected tumors, IONP-DTMs showed 6.4 times higher accumulation in the magnet-treated animals (Figure 12). These results demonstrated the potential use of magnetically guided diatom microcarriers for drug delivery applications.

**Figure 13:**
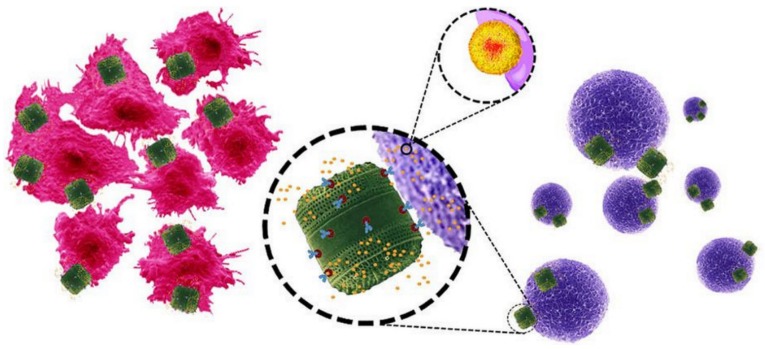
Genetically engineered diatom biosilica (green) containing liposome-encapsulated drug molecules (yellow) can be targeted to both adherent neuroblastoma cells (red) and lymphocyte cells in suspension (purple) by functionalizing the biosilica surface with cell-specific antibodies. Liposome-encapsulated drug molecules are released from the biosilica carrier in the immediate vicinity of the target cells (inset). Reprinted from [70], copyright (2015), with permission from Springer Nature.

In 2015, Javalkote et al. reported the fabrication of magnetically responsive diatoms by two different techniques [82]. The first technique was a simple mixing of pure diatoms with both a drug solution and a ferrofluid (a fluid containing ferromagnetic nanoparticles) leading to the loading of both items in the diatoms in one pot. For the second technique, prior to the drug loading, the diatoms were soaked in a solution containing different ferrous salts followed by addition of ammonia, which promoted the growth of iron oxide nanoparticles directly inside the diatoms. The magnetically active diatoms were demonstrated as able to be guided by the application of an external magnetic field. The magnetically active diatoms were loaded with curcumin as a water insoluble drug model and tested on human cervical cancer (HeLa) cells. In 2015, Delalat et al. reported the use of genetically engineered diatom biosilica as a form of targeting drug-delivery vehicles (Figure 13) [70]. By incorporating the gene domain of protein GB1 in the genome of diatom *Thalassiosira pseudonana*, the authors designed a biosilica material able to bind immunoglobulin G (IgG) antibodies on their surface. The authors labelled these new functionalizable diatoms with rituximab— an antibody specific for the antigen CD20—and demonstrated the specific targeting of cancer cells expressing antigen CD20 both in the case of surface-attached and suspended cells. To overcome the problem of antibody denaturation in organic solvent, the drug was loaded in positively charged nanocapsules as liposomes or micelles before adsorbing them on the negatively charged surfaces of antibody-labelled biosilica. It was shown that both drug-loaded nanocapsules could deliver their cargo (camptothecin and its derivative 7-ethyl-10-hydroxy-camptothecin, also called SN38) at higher levels than the minimum toxic dose after 16 h in DMEM medium. In vitro tests assessed the specific cytotoxicity of SN38 micelle-loaded anti-p75NTR-GB-biosilica towards SH-SY5Y neuroblastoma cells (only 10% of which remained viable after 2 days) with BSR cells as control (95% remaining viable after 2 days). In vivo tests in nude mice further confirmed the efficiency and the reduction of tumor growth after a single dose injection.

**Figure 14:**
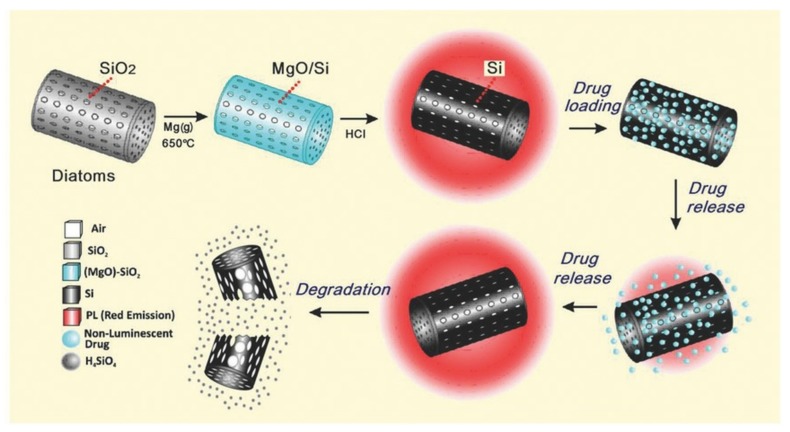
Schematic illustration of the gas/silica displacement method on the basis of the magnesiothermic reduction process employed to convert silica diatoms’ 3D structure into luminescent silicon replicas as a new biodegradable microcarrier with self-reporting function and a unique biological-derived shape that is desirable for high drug loading and sustainable release for broad drug delivery applications. Reproduced from [55], copyright (2015), with permission from John Wiley and Sons.

Maher et al. reported the magnesiothermic reduction process of silica diatom frustules to silicon replicas (Figure 14) [55]. The process improves the specific surface area of the material 13-fold. Moreover, silicon replicas (SiNPs) showed increased biodegradability compared to the silica diatom precursors. While less than 1% of the silica diatoms degraded after 30 days, 20% of the SiNPs were dissolved after the same time in PBS at pH 7.4, 37 °C. Drug loading and release from silica diatoms or SiNPs using daunorubicin as a drug model (an anticancer also used in the treatment of vitreoretinopathy [83]) indicates a better penetration of the drug in the SiNPs, in which the pores are slightly bigger than in the silica diatoms. The study showed that the drug release from SiNPs was partially due to degradation of the particles and not only due to the concentration gradient. The authors also demonstrated for the first time that diatoms could act as self-reporting nanocarriers for both luminescent and non-luminescent drugs by following the photoluminescence (PL) spectrum of the characteristic red band at 682 nm belonging to SiNPs. These were shown to not be toxic towards Raw 264.7 murine macrophage and MDA-MB-231 breast cancer cells [56]. When loaded with doxorubicin, they sustain its release over a period of 30 days and enhance drug cytotoxicity when compared with equivalent free drug concentration, further validating the applications of SiNPs as drug nanocarriers for chemotherapeutics.

More recently, Janićijević and co-workers reported a study on diatomite inorganically modified with aluminum salt that showed potentiation of the ibuprofen antihyperalgesic effect and demonstrated a greater effectiveness of the modified diatomite-ibuprofen composite than the equivalent doses of pure ibuprofen in pain suppression in rats [71]. 

## 5. Diatom Composite Formulations in Drug Delivery Systems

The use of composite formulations made of diatoms mixed with other materials in order to improve, modify, or finely tune their chemical and physical properties has been recently investigated.

**Figure 15:**
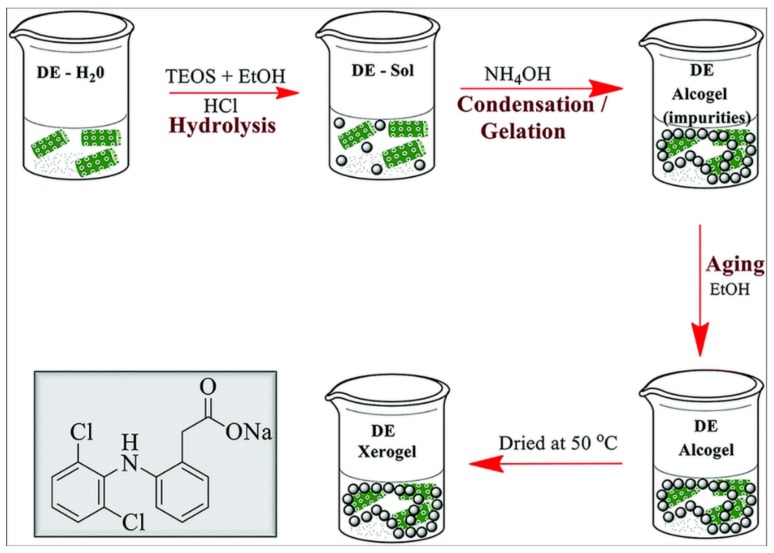
Schematic diagram showing DE-xerogel (DE-XER) synthesis. Inset: chemical structure of diclofenac sodium drug. Reproduced from [84] with permission from the Centre National de la Recherche Scientifique (CNRS) and The Royal Society of Chemistry.

In 2018, Uthappa et al. investigated the use of a novel hybrid material made of diatoms modified with silica xerogel, covalently linked to the surface of DE microparticles (Figure 15) [84]. Dicoflenac, a nonsteroidal anti-inflammatory drug (NSAID) used to treat inflammatory diseases, was chosen as a drug model in a study showing that drug loading and sustained release properties are considerably improved in the xerogel-DE material when compared with neat DE. Interestingly, xerogel-DE is capable of dicoflenac-sustained release, and it is stable in acidic condition, mimicking the human gastric environment over a 36 day period.

Also in 2018, López-Cebral et al. reported the synthesis of a composite material made of diatomaceous earth incorporated in β-chitosan membranes [85]. The formulation benefits from both the mucoadhesive properties of the chitosan membranes and the drug loading and releasing capacities of diatomaceous earth to perform the delivery of the drug by sublingual application. The sublingual pathway is an important alternative for drug administration in patients suffering from oral administration inconveniences such as dysphagia and mucoadhesion, and sustained drug delivery could overcome the difficulties induced by the administration of poorly water-soluble drugs. In their study, López-Cebral and co-workers selected gentamicin and dexamethanose as hydrophilic and hydrophobic drug models, respectively. Increased wettability as well as higher values of surface energy that promotes adhesion to the cells were reported for the new material as a function of the increasing DE percentage within the formulation. Whereas drug loading and release tests of the hydrophilic gentamicin did not show differences between pure β-chitosan and DE-modified membranes, the same experiments with the hydrophobic dexamethanose revealed higher loading capacities and longer sustained and higher dose release from the DE-modified β-chitosan membrane when compared with the pure β-chitosan membrane.

## 6. Conclusions

Diatoms are a remarkable source of highly 3D structured porous material. With many different classes and species, this natural product offers a wide panel of choice for specific applications in science and nanotechnologies. Throughout this review, the potential of diatoms used in drug delivery applications was outlined. Indeed, besides their highly structured shapes, these microparticles offer the advantages of wide surface areas, great mechanical resistances, as well as biodegradability and biocompatibility, making them a very valuable material in drug delivery applications. This promising, inexpensive, eco-friendly, and easily accessible biomaterial complements the technology of synthetic mesoporous silica particles. The surface functionalization of diatoms provides a unique opportunity to enhance their physical and chemical properties, such as drug loading efficiency, sustained and controllable drug release capacities, and targeting delivery potential. In addition, the use of diatoms in composite materials of different ingredients, capable of improving both their physical and chemical properties, was also presented. Even if additional studies highlight the biocompatibility and non-toxicity of diatomite biosilicas, efforts need to be made to undoubtedly demonstrate their potential use in the field of drug delivery and in other medicinal applications. This may be achieved via additional animal studies on long-term toxicity.

## Figures and Tables

**Table 1:** Different diatomite functionalizations and drug-loading (DL) capacities in drug delivery applications.

Diatom sp.	Functionalization		Drug			Ref.
	Aim	Composition	wt%	Model	Class	
***Aulacoseira*** **sp.**	Magnetic guide, drug loading and release	DOPA/Fe_3_O_4_	28	Indomethacin	NSAID	[63]
*Aulacoseira* sp.	Drug loading and release	APTES	22 (I)	Indomethacin (I) Gentamicin (G)	NSAID (I) Antibio (G)	[64]
			15 (G)			
		2-CEPA	24 (I)			
			16 (G)			
		16-PHA	14 (I)			
			22 (G)			
		OTS	14 (I)			
		GPTMS	19 (I)			
		mPEG-silane	17 (I)			
*Aulacoseira* sp.	Drug loading and release	GO	28.5	Indomethacin	NSAID	[65]
n.i.	Tracker nanosized diatomite Tumor targeting delivery	Rhodamine	-	-	-	[66]
*Aulacoseira* sp.	Drug loading and release	APTES	19	Indomethacin	NSAID	[67]
		AEAPTMS	24			
		2-Phos	22			
		16-Phos	15			
n.i.	Tumor targeting delivery	peptide/siRNA	-	siRNA	Gene silencer	[36,38]
*Thalassiosira weissflogii*	Reactive oxygen species (ROS) scavenger	TEMPO	2	Ciprofloxacin	Antibio	[68]
*Aulacoseira* sp.	Temperature-responsive drug release	(O(EG)MA) copolymers	-	Levoflaxin	Antibio	[69]
*Thalassiosira pseudonana*	Tumor targeting delivery	Antibodies	-	Camptothecin and derivatives	Anticancer	[70]
n.i.	Tumor targeting delivery	PEG-CPP	22	Sorafenib	Anticancer	[37]
n.i.	Drug loading and release	Al_2_(SO_4_)_3_	20	Ibuprofen	NSAID	[71]
*Amphora subtropica*	Drug loading and release	Chitosan	-	Doxorubicin	Anticancer	[72]
*Aulacoseira* sp.	Tumor targeting delivery	Vitamin B_12_	6	Cisplatin	Anticancer	[73]
			10	5-FU		
			2	Ruthenium complex		

Not identified (n.i.); dopamine (DOPA); (3-aminopropyl)triethoxysilane (APTES); 2-carboxyethyl phosphonic acid (2-CEPA or 2-phos); 16-phosphono-hexadecanoic acid (16-PHA or 16-phos); 7-octadecyltrichlorosilane (OTS); 3-(glycidyloxypropyl)trimethoxysilane (GPTMS); methoxy-poly(ethyleneglycol)-silane (mPEG-silane); graphene oxide (GO); N-(3-(trimethoxylsilyl)propyl)ethylenediamine (AEAPTMS); (2,2,6,6-tetramethylpiperidin-1-yl)oxy (TEMPO); oligo(ethyleneglycol) methacrylates (O(EG)MA); cell-penetrating peptide PEG derivative (PEG-CPP); 5-fluorouracil (5-FU); antibiotic (Antibio); nonsteroidal anti-inflammatory drug (NSAID).
